# Downregulation of drug transport and metabolism in mice bearing extra-hepatic malignancies

**DOI:** 10.1038/sj.bjc.6604101

**Published:** 2007-12-04

**Authors:** R Sharma, M Kacevska, R London, S J Clarke, C Liddle, G Robertson

**Affiliations:** 1 Storr Liver Unit, Westmead Millennium Institute, University of Sydney, Sydney, New South Wales, Australia; 2 Department of Medicine, University of Sydney, Sydney, New South Wales, Australia; 3 Cancer Pharmacology Unit, ANZAC Research Institute, Concord RG Hospital, Concord, New South Wales, Australia

**Keywords:** CYP3A, drug metabolism, malignancy, inflammation, hepatic transporters

## Abstract

There is increasing evidence of a systemic inflammatory response associated with malignancy, which may have an impact on both drug disposition and resistance to cytotoxic therapy. The impact of inflammation on drug disposition was studied in mice bearing a number of common tumour xenografts. C57BL/6 mice were inoculated with tumour xenografts. Hepatic expressions of Cyp3a and drug transporters were analysed at the mRNA, protein and functional levels (Cyp3a only). Circulating serum cytokines and the hepatic expression of acute phase proteins (APPs) were measured. Intratumoral levels of multidrug resistance genes were determined. Tumour xenografts elicited an inflammatory response that coincided with repression in hepatic Cyp3a11 activity and the expression of a number of hepatic drug transporters. With tumour growth, a progressive reduction in hepatic Cyp3a11 mRNA expression was seen. Conversely, an increase in the hepatic APP expression and circulating interleukin (IL)-6 levels was observed. Furthermore, a correlation was seen between increased intratumoral expression of the multidrug resistance gene, *Mdr1a*, and levels of circulating IL-6. Malignancy results in reduced hepatic drug disposition that correlates with an associated inflammatory response. Reduction of inflammation may improve the clinical outcome for patients receiving chemotherapeutic agents that undergo hepatic metabolism.

The pharmacokinetics (PKs) of chemotherapeutic drugs vary considerably between patients, and because of the narrow therapeutic index of most anticancer agents, results in their inherent lack of safety. Variability in the PKs of anticancer agents can be attributed to a number of factors including comedications, environmental factors, genetic polymorphisms and certain disease states. These factors can have an impact on drug disposition, resulting in either ineffective drug doses or excessive toxicity (Thummel and Wilkinson, 1998; Schuetz, 2004). This concept is supported by a study demonstrating that reductions in CYP3A4 activity in patients with advanced cancer were correlated to an increased plasma concentration of the inflammatory mediators interleukin (IL-)6 and C-reactive protein (CRP) (Rivory *et al*, 2002; Slaviero *et al*, 2003). This was associated with reduced clearance and increased toxicity from docetaxel, a well-characterised substrate for CYP3A4.

The presence of a tumour-induced inflammatory response is increasingly being recognised in patients with solid tumours (Mahmoud and Rivera, 2002; Assenat *et al*, 2006). Clinically, tumour-induced inflammation is characterised by weight loss, night sweats and fevers, as exemplified by the B symptoms associated with lymphoma. Furthermore, the presence of systemic inflammation is recognised as a predictor of worse outcome in patients with malignancy. Raised levels of CRP, an acute phase protein (APP), and proinflammatory cytokines, especially IL-6, are strong, independent prognostic factors for survival in a number of tumour types including breast, renal cell, prostate and colorectal cancers (Jamieson *et al*, 2005; Al Murri *et al*, 2006; Crozier *et al*, 2006; Lamb *et al*, 2006; McArdle *et al*, 2006). IL-6 has further been implicated in promotion of tumour growth, invasion and metastasis, and the development of drug resistance (Kato *et al*, 1998; Esper and Harb, 2005).

The relationship between inflammation and repression of *P*450 activity, in particular CYP3A4, has been extensively studied both *in vitro* and *in vivo* (Chen *et al*, 1994; Carcillo *et al*, 2003; Haack *et al*, 2003). These studies consistently illustrate reduced hepatic expression of CYPs in the presence of acute inflammation, mediated primarily by the proinflammatory cytokines IL-6, IL-1*β* and tumour necrosis factor-*α* (TNF-*α*), resulting in reduced drug metabolism and clearance (Morgan, 1997, 2001; Fang *et al*, 2004).

More recently, acute inflammation has been shown to also impact on the expression and activity of hepatic transporters. Uptake of drugs from the basolateral surface of the hepatocytes into the cell is facilitated by the Na^+^-taurocholate cotransporting polypeptide (NTCP) and organic anion-transporting polypeptide (OATP) families. Following metabolism, efflux of metabolites across the canalicular surface of the hepatocyte into bile is facilitated by members of the ABC family of transporters, specifically the bile salt export pump (Bsep), multidrug resistance-associated protein (MRP2) and members of the multidrug resistance (MDR) family. Efflux across the basolateral surface of the hepatocyte back into the circulation is mediated by MRP3 and MRP4 (Kullak-Ublick *et al*, 2004; Maher *et al*, 2005). These transporters are responsible for the uptake and elimination of a number of chemotherapeutic agents and their disruption may lead to an increased risk of adverse events (Chandra and Brouwer, 2004).

Cytokines, in particular IL-6, reduce the mRNA and protein expressions of some hepatic transporters *in vivo*, and function *in vitro* (Hartmann *et al*, 2001; Lee and Piquette-Miller, 2001; Sukhai *et al*, 2001; Hartmann *et al*, 2002). Conversely, in tumours, studies have demonstrated an increase in the expression of some ABC superfamily transporters on tumour cells in response to IL-6, where they produce the multidrug-resistant tumour phenotype (Conze *et al*, 2001; Duan *et al*, 2002).

In the current study, we have tested the hypothesis that a reduction in both hepatic Cyp3a11 and hepatic transporter expression occurs in the presence of cancer, and is a result of a cytokine release associated with extra-hepatic malignancy. Furthermore, we investigated the effect of tumour-induced inflammation on the expression of multidrug-resistant tranporters within the tumour tissue itself.

## MATERIALS AND METHODS

### Animal experimentation

All animal protocols and studies were conducted in accordance with the guidelines of the Australian Council on Animal Care and approval was obtained from the Westmead Hospital Animal Ethics Committee before commencement of experimentation. C57BL/6 mice were obtained from the Animal Resources Centre, Perth, Western Australia. Animals were kept in a temperature-controlled facility with 12-h light/dark cycles and were fed a standard chow diet and allowed water *ad libitum*. The condition of the animals was assessed daily by trained animal handlers to ensure that they remained well and active during the course of the experimentation.

Eight- to ten-week-old male mice were aseptically inoculated with either 0.3 ml suspension of Englebreth-Holm-Swarm (EHS) sarcoma into the right quadriceps muscle, 3.8 × 10^6^ B16 melanoma cells (gift from Peter Parson, Queensland Institute of Medical Research, Brisbane, QLD, Australia) or 3.8 × 10^6^ E0771 breast cancer cells (gift from Robin Andersen, Peter MacCallum Cancer Centre, Melbourne, VIC, Australia) suspended in 0.3 ml of PBS subcutaneously into the right flank. Control animals were inoculated with the vehicle alone.

Mice were weighed and calliper measurements of the subcutaneous tumour were made daily. The volume of tumour (in mm^3^) was calculated using the equation volume=0.5 × (width × breadth × height). Tumour mass reached approximately 3 g or 10% of total body weight after approximately 3 weeks. Mice were anaesthetised with ketamine and xylazine (30 and 8 *μ*g ml^−1^, respectively in water) before cardiac puncture for the collection of blood. The liver was immediately harvested and snap-frozen in liquid nitrogen and stored at −70°C. Serum was snap-frozen in aliquots in liquid nitrogen and then stored at −70°C. For the time-course experiments, mice inoculated with the EHS sarcoma were harvested at four different time points: days 4, 7, 11 and 18.

### Determination of mRNA expression

Total RNA was isolated from mouse liver and tumour using Trizol reagent (Invitrogen, Mulgrave, Australia) and treated with DNase I (Ambion, Austin, TX, USA) according to the manufacturer's protocols. Complementary DNA was synthesized from 5 *μ*g of total RNA with SuperScript III cDNA First-Strand Synthesis System (Invitrogen) using random hexamer primers. Taqman or SYBR green protocols were used to amplify cDNAs of interest by real-time quantitative PCR (Q-PCR) using a Rotor-Gene 3000 (Corbett Research, Sydney, NSW, Australia). Primers were obtained from Invitrogen and primer/Taqman probes from Applied Biosystems (Forster City, CA, USA). Messenger RNA levels were normalised to 18S mRNA expression. Normalisation to *18S*, *B2M* and *C36B4* mRNA housekeeping genes gave comparable results. Primer and probe sequences are available on request.

### Western blot analysis

Extraction and preparation of reduced proteins from liver tissue were performed as described previously (Ip *et al*, 2003). For immunoblotting, 15 *μ*g of protein was loaded and resolved on 7% sodium dodecyl sulphate–polyacrylamide gel electrophoresis (SDS–PAGE) for 2 h under reducing conditions and then transferred to polyvinylidine difluoride membranes. Membranes containing transporter proteins were then cut into half and the upper portion (mol. wt. >72 kDa) was incubated with either cMOAT/MRP2 (clone M_2_III6) antibody (Signet, Debham, MA, USA) or MRP3 (clone M3II-9) antibody (Signet), followed by a peroxidase-conjugated secondary antibody (Sigma-Aldrich, Castle Hill, NSW, Australia). To control for variability in protein loading, the lower portion of the membrane (mol. wt. <72 kDa) was incubated with the anti-*β*-actin clone AC15 antibody (Sigma-Aldrich), followed by the secondary antibody. Mouse Cyp3a proteins were detected with a polyclonal rabbit anti-rat Cyp3a antibody (Chemikalien, Gertenbach, Germany). Proteins were visualised by SuperSignal West Pico chemiluminescence kit (Pierce Endogen, Rockford, IL, USA) and quantified using densitometric analysis.

### Midazolam sleep test

To determine whether the transcriptional repression of murine *Cyp3a* genes resulted in altered Cyp3a function, activity was assessed using the midazolam sleep time, a specific substrate for Cyp3a enzymes (Watanabe *et al*, 1998). Before harvesting, tumour-bearing and control mice were injected intraperitoneally with midazolam (12 mg kg^−1^). Mice were deemed to be asleep when loss of righting reflex was observed, and awake when this reflex returned.

### Measurement of IL-1*β*, TNF-*α* and IL-6 levels

Cytokine levels in the serum of tumour-bearing and control mice were measured using the Quantikine high-sensitivity mouse IL-1*β*, TNF-*α* and IL-6 immunoassays as per the manufacturer's instructions (R&D Systems Europe, Abingdon, Oxon, UK).

### Statistical analysis

All studies were performed using groups of *n*⩾4 mice. Differences between tumour-bearing and non-tumour-bearing mice were determined by one-way analysis of variance and logistic regression analysis, with *P*<0.05 considered to be statistically significant. Data were log-transformed before analysis in order to stabilise variance if deemed necessary. Analysis was performed using SPSS version 11.5 (SPSS Inc., Chicago, IL, USA).

## RESULTS

### Impact of tumour on Cyp3a11 expression and function

To determine whether the presence of extra-hepatic malignancy affects the activity of hepatic Cyp3a11, mice were injected with a number of tumour xenografts or vehicle. Following tumour inoculation, mice maintained good health and activity levels with no net weight loss observed. No adverse events were noted during the study period and no mice died during the experiment. The time to harvest from tumour implantation was as follows melanoma B16, 20 days; breast E0771, 16 days; and EHS sarcoma, 24 days. The average tumour volume of each tumour type at harvest was as follows melanoma B16, 2290 mm^3^ (2216–3596 mm^3^); breast E0771, 2177 mm^3^ (228–7399 mm^3^); and EHS sarcoma, 4690 mm^3^ (3188–6459 mm^3^).

**Figure 1:**
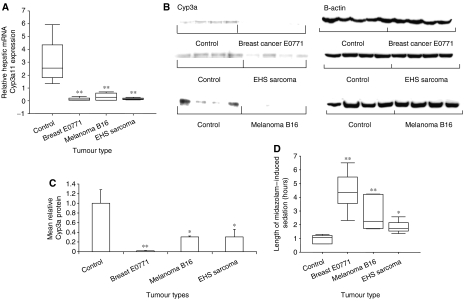
Effect of extra-hepatic malignancy on hepatic Cyp3a11 mRNA expression and Cyp3a protein and activity *in vivo*. Mice (*n*=4–5 per group) were injected with vehicle (control), breast E0771, melanoma B16 or EHS sarcoma xenografts as described in Materials and Methods and killed after 3 weeks, or when the viability of the mouse was threatened. (**A**) Relative quantification of hepatic *Cyp3a11* gene expression in tumour-bearing and control mice. Hepatic Cyp3a11 mRNA levels were determined by Q-PCR and normalised to 18S RNA gene expression. (**B**, **C**) Quantification of Cyp3a protein in the liver of tumour-bearing and control mice by western blot analysis using a rabbit anti-rat Cyp3a antibody. Cyp3a protein expression was quantified by densitometry and then normalised to *β*-actin protein expression. (**D**) Measurement of Cyp3a-mediated metabolism of midazolam in tumour-bearing and control mice. Mice were administered 12 mg kg^−1^ midazolam (i.p.) and the period of sedation was measured. Box and whisker plot, median and interquartile range. Columns, average of four or five mice; bars, s.e.m. ^*^P<0.05; ^**^P<0.001.

On Q-PCR, the presence of malignancy resulted in a significant repression in hepatic Cyp3a11 mRNA compared with controls for all tumour types (Figure 1A). These results were confirmed at the protein level by western analysis (Figure 1B and C). To determine whether repression of Cyp3a11 resulted in altered Cyp3a-mediated drug metabolism, midazolam-induced sleeping times were measured. Significantly prolonged sleeping times were observed for all tumour types compared with controls. In particular, mice bearing implanted breast E0771 cells slept 4.6 times longer, and melanoma B16-bearing mice slept 3 times longer than controls (Figure 1D).

### Evaluation of tumour-induced inflammation

**Figure 2:**
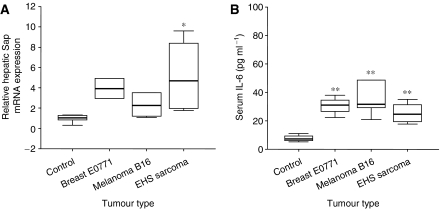
Assessment of inflammation in mice bearing tumour xenografts. Mice (*n*=4–5 per group) were injected with (vehicle) control or breast E0771, melanoma B16 or EHS sarcoma xenografts as described in Materials and Methods. (**A**) Relative quantification of hepatic acute phase protein, Sap, in tumour-bearing and control mice. Hepatic Sap mRNA levels were determined by Q-PCR and normalised to 18S RNA expression. (**B**) Serum concentration of IL-6 in tumour-bearing and control mice. Sera were analysed for IL-6 concentrations by ELISA. Box and whisker plot, median and interquartile range. ^*^P<0.05; ^**^P<0.001.

As described previously, a systemic inflammatory response has been shown to be associated with the repression of Cyp3a activity (Shedlofsky *et al*, 1994; Mayo *et al*, 2000; Frye *et al*, 2002). In order to determine whether the presence of malignancy induced a systemic inflammatory response, the mRNA expression of the hepatic acute phase reactants, serum amyloid protein (Sap) and metallothionein were measured in tumour-bearing mice compared with controls. Additionally, the cytokines contributing to this proinflammatory state were determined. Implantation of EHS sarcoma resulted in a significant increase in mRNA expression of both acute phase genes compared with controls (Figure 2A and B). A significant increase in metallothionein mRNA expression was observed in mice bearing the breast cancer and melanoma xenografts. A trend was observed for the upregulation of Sap in mice bearing melanoma (*P*=0.08) and breast cancer (*P*=0.06) xenografts compared with controls. The proinflammatory cytokines IL-1*β*, TNF-*α* and IL-6 were quantified using ELISA. No significant differences were observed in the circulating levels of IL-1*β* and TNF-*α* between control and tumour-bearing mice (data not shown). However, the level of IL-6 was significantly elevated in all tumour models compared with controls (Figure 2B).

### Regulation of hepatic transporter genes in the presence of extra-hepatic malignancy

**Figure 3:**
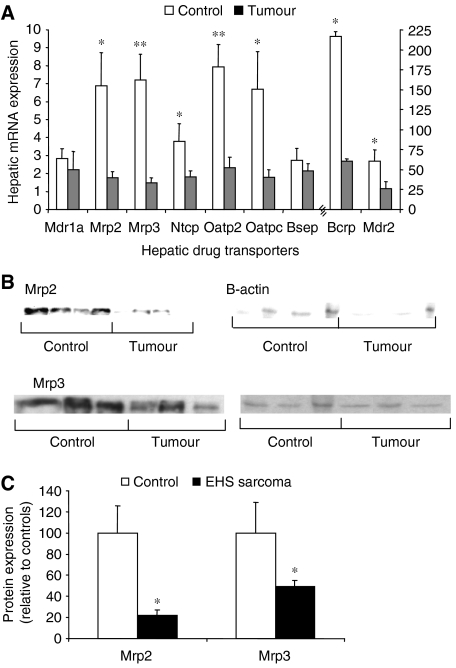
Effect of extra-hepatic malignancy on expression of hepatic drug transporters and Mrp2 and Mrp3 protein expression *in vivo*. Mice (*n*=4–6 per group) were injected with vehicle control or EHS sarcoma and killed after 3 weeks or when the viability of the mouse was threatened as described in Materials and Methods. (**A**) Quantification of hepatic transporter gene expression in tumour-bearing and control mice. mRNA expression of hepatic drug transporters was determined by Q-PCR and normalised to 18S RNA mRNA expression. (**B**, **C**) Quantification of Mrp2 and Mrp3 proteins in the liver of tumour-bearing and control mice by western blot analysis. Expression of Mrp2 and Mrp3 proteins was quantified by densitometry and then normalised to *β*-actin protein expression. Columns, average of five or six mice; bars, s.e.m. ^*^P<0.05; ^**^P<0.001.

As acute inflammation represses the expression of hepatic transporters, the expression levels of hepatic transporters in the presence of tumour-induced inflammation were determined (Hartmann *et al*, 2001, 2002; Lee and Piquette-Miller, 2001; Sukhai *et al*, 2001). The mRNA expression levels of relevant transporters were determined in mice bearing EHS sarcoma tumours and compared with controls by Q-PCR. In tumour-bearing mice, the hepatic expression levels of Mdr2, Mrp2, Mrp3, Ntcp, Oatp2, Oatp-c and Bcrp were significantly reduced compared with controls (Figure 3A). The expression of Mdr1a and Bsep was not significantly altered. Immunoblotting confirmed the repression of Mrp2 and Mrp3 at the protein level (Figure 3B and C).

### Relationship between tumour growth and diminished Cyp3a11 expression

**Figure 4:**
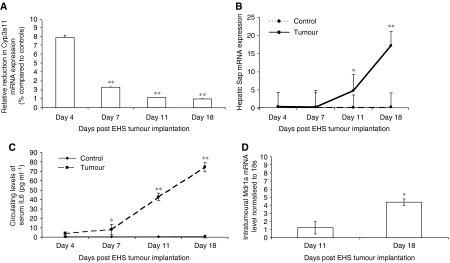
Time-course study examining the impact of tumour growth on hepatic drug metabolism, tumour drug disposition and hepatic inflammatory response. Mice (*n*=4–6) were injected with (vehicle) control or EHS sarcoma as described in Materials and Methods and killed at days 4, 7, 11 and 18. (**A**) Relative quantification of hepatic Cyp3a11 expression in tumour-bearing mice compared with control mice at four different time points. Hepatic Cyp3a11 mRNA levels were determined by Q-PCR and normalised to 18S RNA gene expression. (**B**) Relative quantification of hepatic Sap expression in tumour-bearing and control mice at four different time points. The expression of hepatic Sap was determined by Q-PCR and normalised to 18S RNA mRNA. (**C**) Serum concentration of IL-6 in tumour-bearing and control mice at advancing time points. Sera were analysed for IL-6 concentrations by ELISA. (**D**) Relative quantification of Mdr1a gene expression within the implanted EHS tumour at days 11 and 18. Tumoral Mdr1a mRNA expression was determined by Q-PCR and normalised to 18S RNA gene expression. These time points were chosen due to the small size of the tumour mass before day 11. Points, average of 4–6 mice; bars, s.e.m., ^*^P<0.05; ^**^P<0.001.

In order to assess whether the repression of hepatic Cyp3a11 mRNA expression and the presence of inflammation were dependent on tumour growth or the presence of malignancy *per se*, mice were injected with EHS sarcoma and harvested at different time points. A progressive decrease in the relative expression of hepatic Cyp3a11 mRNA levels was observed at days 4, 7, 11 and 18 between control and tumour-bearing mice (Figure 4A). A significant increase in the hepatic mRNA expression of Sap was also observed at each time point in tumour-bearing mice compared with controls (Figure 4B). Furthermore, a significant increase was seen in circulating IL-6 levels in the serum of tumour-bearing mice compared with controls at days 7, 11 and 18 (Figure 4C). A statistically significant positive rank correlation of 0.6 was observed between circulating IL-6 levels and Sap. A trend was observed between increasing serum levels of IL-6 and reduced hepatic Cyp3a11 mRNA expression (*R*=−0.407, *P*=0.07).

### Impact of increasing tumour growth on drug transporter expression

As administration of exogenous IL-6 to cancer cells *in vitro* induces drug resistance, the expression of multidrug resistance genes was assessed within the tumour at different time points (Borsellino *et al*, 1995; Conze *et al*, 2001). A significant increase in the mRNA levels of the multidrug-resistance transporter, Mdr1a, was observed within the tumour tissue at day 18 compared with day 11 (Figure 4D). This was associated with increasing levels of both circulating IL-6 (*P*<0.05; 95% CI: 1.2–2.9) and mRNA expression levels of IL-6 within the tumour itself (*P*<0.05; 95% CI: 1.0–2.6). Combined with the observed decrease in expression of MRPs and other ABC transporters in the livers of tumour-bearing mice (Figure 2A), this finding highlights the divergent regulation of drug transporters in cancerous tissues compared with other organs in the body. No change was observed in the intratumoral mRNA expression of Bcrp, Mdr2, Mrp2 and Mrp3 (data not shown).

## DISCUSSION

Alterations in the expression of enzymes and transport proteins involved in hepatic drug disposition can have an important influence on the susceptibility of organs and tissues to the therapeutic and toxic effects of anticancer drugs. The first aim of this study, therefore, was to assess the effect of extra-hepatic malignancy on hepatic Cyp3a mRNA expression and activity, and whether this was associated with a tumour-induced acute phase response.

We have shown in murine models of a number of common malignancies that there is a significant repression of hepatic Cyp3a11 mRNA expression in tumour-bearing mice compared with controls, functionally reflected by a prolongation of the sedative effect of midazolam, a CYP3A substrate. This study further expands on previous experiments from our group in which a transgenic mouse model of human CYP3A4 regulation was used to demonstrate that the presence of tumour resulted in downregulation of the CYP3A4 transgene, which was linked to a systemic acute phase response. Furthermore, IL-6 was localised to the tumour and not the liver in tumour-bearing animals, suggesting that the tumour or its associated stroma is the source of IL-6. As a result of this study, a mechanistic link was suggested between tumour-derived cytokines and impaired drug metabolism (Slaviero *et al*, 2006). While repression of hepatic Cyp3a11 at the mRNA, protein and functional level was found in majority of tumour models in this study, the tumour-associated inflammatory response was not equal between these models. This was not an unexpected result. It is well described that certain malignancies, including ovarian, myeloma and renal cell carcinoma, elicit a more pronounced inflammatory response than other tumours and this may account for the differences in the cytokine profiles observed (Nash *et al*, 1999). The hepatic mRNA expression levels of Sap exhibited a varied response and this suggests that altered hepatic gene regulation in the presence of malignancy is complex, and that the genes affected by inflammation associated with malignancy may be impacted upon by divergent cytokine profiles and regulatory mechanisms.

While the cytokines contributing to the systemic inflammatory response associated with malignancy have not been extensively characterised, the cytokines involved in the repression of murine Cyp3a11 in acute inflammation have been extensively studied both *in vitro* and *in vivo*. *In vitro* studies in hepatoma cell lines illustrate the downregulation of Cyp3a11 exposed to the proinflammatory cytokines IL-6, IL-1*β* and TNF-*α* (Lee and Piquette-Miller, 2001; Sukhai *et al*, 2001). Furthermore, the inducible expression of CYP3A4 by rifampicin in human primary hepatocytes in culture is abrogated by the administration of IL-6, suggesting a central role of IL-6 in the repression of CYP3A (Muntane-Relat *et al*, 1995; Guillen *et al*, 1998). On administration of these cytokines to rodents, reproducible reductions of Cyp3a mRNA expression with IL-1*β*, TNF-*α* and especially IL-6 were observed (Ghezzi *et al*, 1986a, 1986b; Ferrari *et al*, 1993). Furthermore, in *in vivo* studies in IL-6-deficient mice, no repression in Cyp3a11 was seen on treatment with turpentine or tuberculosis vaccine (Siewert *et al*, 2000). Moreover, deletion of the IL-1*β* and TNF-*α* receptors *in vivo* had little or no effect on Cyp3a11 downregulation (Ashino *et al*, 2004). Therefore, from both preclinical and clinical studies, IL-6 appears to play a central role in the downregulation of the CYP3A subfamily of enzymes in response to acute inflammation, consistent with our results (Aitken *et al*, 2006).

The next aim of this study was to assess the impact of tumour-associated inflammation on the expression of hepatic transporters in order to ascertain whether derangement of drug disposition represents a more global phenomena occurring within the liver, involving more than drug metabolism. This is the first report of the involvement of drug transporters in the altered drug response in malignancy, specifically, the transcriptional downregulation of the hepatic mRNA expression of Mdr2, Mrp2, Mrp3, Ntcp, Oatp2, Oatp-c and Bcrp. This represents a widespread repression of drug transport within the liver with transporters mediating both uptake and efflux from the affected hepatocyte. These findings are consistent with previously reported *in vitro* and *in vivo* models of acute inflammation in which IL-6 is reported to be the major contributor to the repression of hepatic transporter mRNA expression (Siewert *et al*, 2000; Hartmann *et al*, 2001; Teng and Piquette-Miller, 2005). The mRNA expression of Mdr1a was not anticipated to be reduced, based on the findings of previous studies that report the importance of post-translational factors in Mdr1a expression (Cornwell, 1991). We did not observe a repression of Bsep mRNA expression in tumour-bearing animals, which was contrary to previous studies in acute inflammation. Bsep expression is reported to be reduced with the administration of IL-6 and endotoxin to mice; however, the mechanism by which Bsep is regulated remains unclear and it is possible that cytokines associated with malignancy may not affect Bsep mRNA expression (Teng and Piquette-Miller, 2005). These findings further emphasise the global repression of hepatic drug disposition in the setting of malignancy and warrant further investigation particularly assessing the functional impact of these transporters on the distribution and elimination of cytotoxic agents within the liver.

The final aspect of this study was to determine the effect of tumour size on Cyp3a expression and plasma cytokine concentrations. We have clearly shown that the transcriptional downregulation of Cyp3a11 mRNA is dependent on tumour growth. This was also true of the acute phase response with progressive increases in hepatic Sap mRNA and levels of circulating IL-6 observed at each advancing time point. Furthermore, we observed an increase in the expression of Mdr1a within the tumour of mice bearing the EHS sarcoma from day 11 to day 18. This was in direct contrast with the mRNA expression of Mdr1a within the liver that is reduced, albeit not significantly. These time-course studies are novel in that they illustrate that the acute phase response and subsequent impaired drug metabolism are dependent on tumour growth. This suggests that the tumour itself, its supporting vasculature and/or stroma may be the source of IL-6. Furthermore, our findings suggest an association between circulating IL-6 levels and the upregulation of multidrug resistance genes within the tumour. This is consistent with *in vitro* studies in which the autocrine production of IL-6 in breast cancer cells was demonstrated to increase MDR expression within the tumor cells themselves (Borsellino *et al*, 1995; Conze *et al*, 2001).

The findings of the present study are unlikely to be the result of the mice being perturbed from the tumour implantation *per se*, as they remained in good health throughout the experiment. We have shown that tumor-bearing animals have evidence of a systemic inflammatory state and that this contributes to a global derangement of hepatic drug handling. Furthermore, we have shown that this is not specific for a particular tumour type but is a general phenomenon that applies in varying degrees to a number of tumour types. This is in the context of increased multidrug resistance gene expression within the tumour itself. While the molecular mechanisms are not well understood, this study raises the possibility that patients with cancer who develop an inflammatory response may have reduced metabolism and tumour chemosensitivity and/or increased toxicity. Serum concentrations of APPs or cytokines could potentially be used as predictive factors for hepatic handling of cytotoxic drugs. In addition, reversal of the inflammatory process before administration of chemotherapy could potentially negate the deranged drug phenotype and improve treatment tolerance and efficacy. These animal models create an experimental platform to assess such reversal strategies that could guide future clinical studies.

Additional studies are required to ascertain whether a correlation exists between reduced expression of hepatic transporters and impaired drug handling *in vivo*. Further work is also needed to investigate the interplay of cytokine signalling pathways with transcription factors known to be relevant to drug-metabolising and transporting genes such as hepatocyte nuclear factor-4*α*, signal transducer and activators of transcription 3, CCAAT enhancer binding proteins and the pregnane X receptor, in order to further understand the mechanism by which IL-6 regulates drug clearance in the presence of tumours (Roy-Chowdhury *et al*, 2003; Tirona *et al*, 2003).

## References

[bib1] Aitken AE, Richardson TA, Morgan ET (2006) Regulation of drug-metabolizing enzymes and transporters in inflammation. Annu Rev Pharmacol Toxicol 46: 123–149 16402901 10.1146/annurev.pharmtox.46.120604.141059

[bib2] Al Murri AM, Bartlett JM, Canney PA, Doughty JC, Wilson C, McMillan DC (2006) Evaluation of an inflammation-based prognostic score (GPS) in patients with metastatic breast cancer. Br J Cancer 94: 227–230 16404432 10.1038/sj.bjc.6602922 PMC2361117

[bib3] Ashino T, Oguro T, Shioda S, Horai R, Asano M, Sekikawa K, Iwakura Y, Numazawa S, Yoshida T (2004) Involvement of interleukin-6 and tumor necrosis factor alpha in CYP3A11 and 2C29 down-regulation by Bacillus Calmette–Guerin and lipopolysaccharide in mouse liver. Drug Metab Dispos 32: 707–714 15205385 10.1124/dmd.32.7.707

[bib4] Assenat E, Gerbal-chaloin S, Maurel P, Vilarem MJ, Pascussi JM (2006) Is nuclear factor kappa-B the missing link between inflammation, cancer and alteration in hepatic drug metabolism in patients with cancer? Eur J Cancer 42: 785–792 16510279 10.1016/j.ejca.2006.01.005

[bib5] Borsellino N, Belldegrun A, Bonavida B (1995) Endogenous interleukin 6 is a resistance factor for *cis*-diamminedichloroplatinum and etoposide-mediated cytotoxicity of human prostate carcinoma cell lines. Cancer Res 55: 4633–4639 7553641

[bib6] Carcillo JA, Doughty L, Kofos D, Frye RF, Kaplan SS, Sasser H, Burckart GJ (2003) Cytochrome *P*450 mediated-drug metabolism is reduced in children with sepsis-induced multiple organ failure. Intensive Care Med 29: 980–984 12698250 10.1007/s00134-003-1758-3

[bib7] Chandra P, Brouwer KL (2004) The complexities of hepatic drug transport: current knowledge and emerging concepts. Pharm Res 21: 719–735 15180326 10.1023/b:pham.0000026420.79421.8f

[bib8] Chen YL, Le Vraux V, Leneveu A, Dreyfus F, Stheneur A, Florentin I, De Sousa M, Giroud JP, Flouvat B, Chauvelot-Moachon L (1994) Acute-phase response, interleukin-6, and alteration of cyclosporine pharmacokinetics. Clin Pharmacol Ther 55: 649–660 8004881 10.1038/clpt.1994.82

[bib9] Conze D, Weiss L, Regen PS, Bhushan A, Weaver D, Johnson P, Rincon M (2001) Autocrine production of interleukin 6 causes multidrug resistance in breast cancer cells. Cancer Res 61: 8851–8858 11751408

[bib10] Cornwell MM (1991) Molecular biology of P-glycoprotein. Cancer Treat Res 57: 37–56 1686722 10.1007/978-1-4615-3872-1_3

[bib11] Crozier JE, McKee RF, McArdle CS, Angerson WJ, Anderson JH, Horgan PG, McMillan DC (2006) The presence of a systemic inflammatory response predicts poorer survival in patients receiving adjuvant 5-FU chemotherapy following potentially curative resection for colorectal cancer. Br J Cancer 94: 1833–1836 16721360 10.1038/sj.bjc.6603185 PMC2361334

[bib12] Duan Z, Lamendola DE, Penson RT, Kronish KM, Seiden MV (2002) Overexpression of IL-6 but not IL-8 increases paclitaxel resistance of U-2OS human osteosarcoma cells. Cytokine 17: 234–242 12027404 10.1006/cyto.2001.1008

[bib13] Esper DH, Harb WA (2005) The cancer cachexia syndrome: a review of metabolic and clinical manifestations. Nutr Clin Pract 20: 369–376 16207677 10.1177/0115426505020004369

[bib14] Fang C, Yoon S, Tindberg N, Jarvelainen HA, Lindros KO, Ingelman-Sundberg M (2004) Hepatic expression of multiple acute phase proteins and down-regulation of nuclear receptors after acute endotoxin exposure. Biochem Pharmacol 67: 1389–1397 15013855 10.1016/j.bcp.2003.12.012

[bib15] Ferrari L, Jouzeau JY, Gillet P, Herber R, Fener P, Batt AM, Netter P (1993) Interleukin-1 beta differentially represses drug-metabolizing enzymes in arthritic female rats. J Pharmacol Exp Ther 264: 1012–1020 8437102

[bib16] Frye RF, Schneider VM, Frye CS, Feldman AM (2002) Plasma levels of TNF-alpha and IL-6 are inversely related to cytochrome *P*450-dependent drug metabolism in patients with congestive heart failure. J Card Fail 8: 315–319 12411982 10.1054/jcaf.2002.127773

[bib17] Ghezzi P, Saccardo B, Bianchi M (1986a) Recombinant tumor necrosis factor depresses cytochrome *P*450-dependent microsomal drug metabolism in mice. Biochem Biophys Res Commun 136: 316–321 3486657 10.1016/0006-291x(86)90912-5

[bib18] Ghezzi P, Saccardo B, Villa P, Rossi V, Bianchi M, Dinarello CA (1986b) Role of interleukin-1 in the depression of liver drug metabolism by endotoxin. Infect Immun 54: 837–840 3491050 10.1128/iai.54.3.837-840.1986 PMC260246

[bib19] Guillen MI, Donato MT, Jover R, Castell JV, Fabra R, Trullenque R, Gomez-Lechon MJ (1998) Oncostatin M down-regulates basal and induced cytochromes *P*450 in human hepatocytes. J Pharmacol Exp Ther 285: 127–134 9536002

[bib20] Haack MJ, Bak ML, Beurskens R, Maes M, Stolk LM, Delespaul PA (2003) Toxic rise of clozapine plasma concentrations in relation to inflammation. Eur Neuropsychopharmacol 13: 381–385 12957337 10.1016/s0924-977x(03)00042-7

[bib21] Hartmann G, Cheung AK, Piquette-Miller M (2002) Inflammatory cytokines, but not bile acids, regulate expression of murine hepatic anion transporters in endotoxemia. J Pharmacol Exp Ther 303: 273–281 12235261 10.1124/jpet.102.039404

[bib22] Hartmann G, Kim H, Piquette-Miller M (2001) Regulation of the hepatic multidrug resistance gene expression by endotoxin and inflammatory cytokines in mice. Int Immunopharmacol 1: 189–199 11360920 10.1016/s0162-3109(00)00271-x

[bib23] Ip E, Farrell GC, Robertson G, Hall P, Kirsch R, Leclercq I (2003) Central role of PPARalpha-dependent hepatic lipid turnover in dietary steatohepatitis in mice. Hepatology 38: 123–132 12829994 10.1053/jhep.2003.50307

[bib24] Jamieson NB, Glen P, McMillan DC, McKay CJ, Foulis AK, Carter R, Imrie CW (2005) Systemic inflammatory response predicts outcome in patients undergoing resection for ductal adenocarcinoma head of pancreas. Br J Cancer 92: 21–23 15597096 10.1038/sj.bjc.6602305 PMC2361749

[bib25] Kato H, Kinoshita T, Suzuki S, Nagasaka T, Hatano S, Murate T, Saito H, Hotta T (1998) Production and effects of interleukin-6 and other cytokines in patients with non-Hodgkin's lymphoma. Leuk Lymphoma 29: 71–79 9638977 10.3109/10428199809058383

[bib26] Kullak-Ublick GA, Stieger B, Meier PJ (2004) Enterohepatic bile salt transporters in normal physiology and liver disease. Gastroenterology 126: 322–342 14699511 10.1053/j.gastro.2003.06.005

[bib27] Lamb GW, McMillan DC, Ramsey S, Aitchison M (2006) The relationship between the preoperative systemic inflammatory response and cancer-specific survival in patients undergoing potentially curative resection for renal clear cell cancer. Br J Cancer 94: 781–784 16523196 10.1038/sj.bjc.6603034 PMC3216422

[bib28] Lee G, Piquette-Miller M (2001) Influence of IL-6 on MDR and MRP-mediated multidrug resistance in human hepatoma cells. Can J Physiol Pharmacol 79: 876–884 11697747

[bib29] Maher JM, Slitt AL, Cherrington NJ, Cheng X, Klaassen CD (2005) Tissue distribution and hepatic and renal ontogeny of the multidrug resistance-associated protein (Mrp) family in mice. Drug Metab Dispos 33: 947–955 15802388 10.1124/dmd.105.003780

[bib30] Mahmoud FA, Rivera NI (2002) The role of C-reactive protein as a prognostic indicator in advanced cancer. Curr Oncol Rep 4: 250–255 11937016 10.1007/s11912-002-0023-1

[bib31] Mayo PR, Skeith K, Russell AS, Jamali F (2000) Decreased dromotropic response to verapamil despite pronounced increased drug concentration in rheumatoid arthritis. Br J Clin Pharmacol 50: 605–613 11136300 10.1046/j.1365-2125.2000.00314.x PMC2015009

[bib32] McArdle PA, Mir K, Almushatat AS, Wallace AM, Underwood MA, McMillan DC (2006) Systemic inflammatory response, prostate-specific antigen and survival in patients with metastatic prostate cancer. Urol Int 77: 127–129 16888416 10.1159/000093905

[bib33] Morgan ET (1997) Regulation of cytochromes P450 during inflammation and infection. Drug Metab Rev 29: 1129–1188 9421688 10.3109/03602539709002246

[bib34] Morgan ET (2001) Regulation of cytochrome *P*450 by inflammatory mediators: why and how? Drug Metab Dispos 29: 207–212 11181485

[bib35] Muntane-Relat J, Ourlin JC, Domergue J, Maurel P (1995) Differential effects of cytokines on the inducible expression of CYP1A1, CYP1A2, and CYP3A4 in human hepatocytes in primary culture. Hepatology 22: 1143–1153 7557864

[bib36] Nash MA, Ferrandina G, Gordinier M, Loercher A, Freedman RS (1999) The role of cytokines in both the normal and malignant ovary. Endocr Relat Cancer 6: 93–107 10732792 10.1677/erc.0.0060093

[bib37] Rivory LP, Slaviero KA, Clarke SJ (2002) Hepatic cytochrome *P*450 3A drug metabolism is reduced in cancer patients who have an acute-phase response. Br J Cancer 87: 277–280 12177794 10.1038/sj.bjc.6600448 PMC2364233

[bib38] Roy-Chowdhury J, Locker J, Roy-Chowdhury N (2003) Nuclear receptors orchestrate detoxification pathways. Dev Cell 4: 607–608 12737794 10.1016/s1534-5807(03)00131-x

[bib39] Schuetz EG (2004) Lessons from the CYP3A4 promoter. Mol Pharmacol 65: 279–281 14742668 10.1124/mol.65.2.279

[bib40] Shedlofsky SI, Israel BC, McClain CJ, Hill DB, Blouin RA (1994) Endotoxin administration to humans inhibits hepatic cytochrome *P*450-mediated drug metabolism. J Clin Invest 94: 2209–2214 7989576 10.1172/JCI117582 PMC330046

[bib41] Siewert E, Bort R, Kluge R, Heinrich PC, Castell J, Jover R (2000) Hepatic cytochrome P450 down-regulation during aseptic inflammation in the mouse is interleukin 6 dependent. Hepatology 32: 49–55 10869288 10.1053/jhep.2000.8532

[bib42] Slaviero KA, Clarke SJ, Rivory LP (2003) Inflammatory response: an unrecognised source of variability in the pharmacokinetics and pharmacodynamics of cancer chemotherapy. Lancet Oncol 4: 224–232 12681266 10.1016/s1470-2045(03)01034-9

[bib43] Slaviero KA, Rivory LP, Brown SL, Liddle C, Clarke SJ, Robertson GR (2006) Transcriptional repression of hepatic CYP3A4 gene in the presence of cancer. Clin Cancer Res 12: 7492–7497 17189422 10.1158/1078-0432.CCR-06-0023

[bib44] Sukhai M, Yong A, Pak A, Piquette-Miller M (2001) Decreased expression of P-glycoprotein in interleukin-1beta and interleukin-6 treated rat hepatocytes. Inflamm Res 50: 362–370 11506391 10.1007/PL00000257

[bib45] Teng S, Piquette-Miller M (2005) The involvement of the pregnane X receptor in hepatic gene regulation during inflammation in mice. J Pharmacol Exp Ther 312: 841–848 15456840 10.1124/jpet.104.076141

[bib46] Thummel KE, Wilkinson GR (1998) *In vitro* and *in vivo* drug interactions involving human CYP3A. Annu Rev Pharmacol Toxicol 38: 389–430 9597161 10.1146/annurev.pharmtox.38.1.389

[bib47] Tirona RG, Lee W, Leake BF, Lan LB, Cline CB, Lamba V, Parviz F, Duncan SA, Inoue Y, Gonzalez FJ, Schuetz EG, Kim RB (2003) The orphan nuclear receptor HNF4alpha determines PXR- and CAR-mediated xenobiotic induction of CYP3A4. Nat Med 9: 220–224 12514743 10.1038/nm815

[bib48] Watanabe M, Tateishi T, Asoh M, Nakura H, Tanaka M, Kumai T, Kobayashi S (1998) Effects of glucocorticoids on pharmacokinetics and pharmacodynamics of midazolam in rats. Life Sci 63: 1685–1692 9806224 10.1016/s0024-3205(98)00440-8

